# Supporting medication reconciliation in primary care: a theory-informed qualitative study in Portugal

**DOI:** 10.1017/S1463423626101200

**Published:** 2026-05-06

**Authors:** Raquel Ascenção, João Costa, Paula Broeiro-Gonçalves

**Affiliations:** 1 Laboratory of Clinical Pharmacology and Therapeutics, Faculdade de Medicina, Universidade de Lisboa, Lisbon, Portugal; 2 Nova Medical School, Universidade Nova de Lisboa, Portugal

**Keywords:** family practice, medication errors/prevention and control, medication reconciliation, primary health care

## Abstract

**Aim:**

To explore behavioural determinants influencing General Practitioner (GP)-led medication reconciliation (MedRec) and inform the development of a theory-informed implementation strategy tailored to the primary care context.

**Background:**

Despite national and international recommendations endorsing MedRec to reduce medication errors, its consistent implementation in primary care remains limited.

**Methods:**

We conducted a qualitative study involving GPs working in the largest Health Region in Portugal, building on findings from preceding quantitative studies. Data were analysed using a Theoretical Domains Framework (TDF)-informed approach. Key determinants were mapped to intervention functions using the Behaviour Change Wheel (BCW), and candidate behavioural change techniques (BCTs) were subsequently proposed.

**Findings:**

A total of 22 GPs participated in three focus group discussions. The ‘Environmental context and resources’ domain gathered the most coded segments, related to patients, other health professionals, electronic health records, and time constraints, mainly reflecting perceived barriers. ‘Knowledge’ and ‘Skills’ emerged as key domains, with ambiguity in the MedRec definition undermining its explicit recognition and influencing other domains. Facilitators included GPs’ commitment to patient safety aligned with GPs’ professional role. The interplay between barriers and facilitators suggested a cascading effect across domains. Candidate BCTs proposed to address these determinants included feedback on behaviour/outcomes, self-monitoring, prompts/cues, restructuring and adding objects to the environment. This study provides a theory-informed foundation for designing tailored implementation strategies to support MedRec practices in Portuguese primary care. Future work should focus on assessing the appropriateness, feasibility and acceptability of the proposed BCTs within the constraints of real-world primary care settings.

## Introduction

Adverse drug reactions (ADR) constitute a significant source of iatrogenic morbidity and mortality in clinical practice, including in the primary care setting (Insani *et al*., 2021). ADR arising from medication errors are preventable, and medication reconciliation (MedRec) is one of several strategies targeted at this unmet need (Pronovost *et al*., 2003; Sponsler *et al*., 2015).

MedRec involves creating the most accurate list possible of all medications a patient is using, including supporting information, such as a history of ADRs, and comparing this list with the current prescriptions (Penm *et al*., 2019). Several organizations, including the World Health Organization (WHO) (World Health Organization, 2019b), endorse MedRec as a key strategy for ensuring that the correct medication is available at all transition points within the healthcare system, thus reducing the probability of medication errors and medication-related harm.

Although MedRec has traditionally been associated with transitions in hospital care, it is increasingly recognized as a necessary safety practice in primary care (ISMP Canada, 2015). General practitioners (GPs) provide longitudinal continuity of care and support patients across formal or informal transitions of care. For example, after a hospital discharge, GPs can identify if the patient continues using a discontinued medicine or whether a chronic medication was unintentionally omitted in the discharge orders. Therefore, GPs are ideally placed to safeguard medication continuity (Jeffers and Baker, 2016) through MedRec.

As part of a broader agenda for medication optimization, MedRec also helps establish an accurate and comprehensive medication list, a prerequisite for informed clinical decision-making and therapeutic reasoning, particularly for strategies such as medication review and deprescribing (World Health Organization, 2019a).

Nonetheless, the implementation of MedRec in primary care remains inconsistent. While previous studies have explored its challenges (Redmond *et al*., 2020; Gionfriddo *et al*., 2021), to the best of our knowledge, none have applied contemporary structured theoretical frameworks to systematically identify behavioural barriers and facilitators that could inform implementation strategies for GP-led MedRec. Yet, theory is recognized as a key component in the development of strategies to address implementation challenges, particularly those involving behaviours deeply embedded in routine clinical workflows, such as MedRec (Skivington *et al*., 2024).

This study aimed to explore the behavioural determinants of MedRec among GPs working in the largest health region in Portugal, using the Theoretical Domains Framework (TDF) to identify barriers and facilitators to its implementation in clinical practice. By focusing on the underlying behavioural contributing factors, this work seeks to inform the development of an implementation strategy tailored to the primary care context, guided by the Behaviour Change Wheel (BCW) Framework.

## Methods

We conducted a qualitative study using the TDF to guide data collection and analysis. The TDF identifies factors influencing health professionals’ behaviours regarding evidence-based recommendations across 14 domains, recognizing cognitive, emotional, social, and environmental influences (Cane *et al*., 2012; Atkins *et al*., 2017). It has been extensively used in research to investigate clinicians’ challenges in adopting evidence-based practices and policies (Dyson and Cowdell, 2021).

The study received approval (Opinion No. 073/CES/INV/2022) from the Ethics Committee for Lisbon and Tagus Valley (LTV) Regional Administration, as part of a broader mixed methods research project aimed at understanding and improving MedRec practices in the LTV Health Region. The preceding quantitative phase, published separately (Ascenção *et al*., 2025), involved two cross-sectional studies designed to characterize GPs’ perceptions, attitudes and practices, and the formal procedures at the Primary Health Care Units [PHCUs] level. Briefly, we found that one in four PCHUs reported having a formal MedRec procedure, and about 30% of GPs (*n* = 58) were unfamiliar with the term. After being introduced to its definition in the survey context, nearly all (*n* = 191; 92%) acknowledged its clinical relevance. Most (*n* = 176; 85%) reported performing MedRec in at least 25% of post-discharge appointments. These findings revealed important gaps in GPs’ practices and unit-level procedures, which informed the design and focus of this qualitative study.

Building on the TDF-based behavioural analysis, we applied the BCW (Michie *et al*., 2014) to map the key behavioural determinants onto relevant intervention functions and candidate behaviour change techniques (BCTs).

### Study setting

The Portuguese National Health Service (NHS) is tax-funded, universal, and primarily free of charge. Yet, it operates alongside the health subsystems and private voluntary health insurance schemes, whose uptake has been steadily rising (Portugal: Country Health Profile, 2023). At the time of this study, five regional health administrations were responsible for executing the national health policy objectives at the local level in mainland Portugal. The LVT Health Region was the largest, providing care for around 4 million inhabitants (Ministério da Saúde, 2025).

Medications are prescribed through a mandatory electronic prescribing system, regardless of whether the prescription originates from the public or private sector and are dispensed through private community pharmacies. MedRec is recommended by the Directorate-General of Health (DGS) and included in the most recent National Patient Safety Plan (2021–2026) (Direção-Geral da Saúde, 2024).

In the NHS, GPs work mostly in PHCUs, either Family Health Units, autonomous multidisciplinary teams responsible for a specific population, or Personalized Healthcare Units, group practices following a pre-2006 reform model with less autonomy (Portugal: Country Health Profile, 2023). GPs usually have personal lists where every patient is allocated a named GP. GPs act as gatekeepers to secondary care and as advocates, holding a central role in managing medications for their patients. MedRec in primary care is predominantly GP-led, with no systematic involvement of clinical pharmacists.

Despite the growth of the primary care network in the past decade, Portugal is facing serious predicaments. In the LVT Health Region, 1 in 4 inhabitants had no access to a named GP as of December 2023 (Ministério da Saúde, 2025). This adds to the complexity of an increasingly aged population, including multimorbidity and polypharmacy (Midão *et al*., 2018).

### Target behaviour

The target behaviour for this study was the performance of MedRec in routine primary care practice. Our analysis focused on the individual-level behaviour of GPs, exploring what influences their MedRec practices through a TDF-informed lens.

In primary care, MedRec may occur in various clinical contexts, most often surrounding transitions of care when medication changes are expected, including post-discharge follow-up and chronic disease management after consultations with other physicians or emergency department visits due to acute events.

The scope of this study was limited to the structured process of MedRec (obtaining and maintaining an accurate and comprehensive medication list, verifying it against available sources, and resolving discrepancies). Broader medication optimization practices, such as comprehensive medication review or deprescribing, were not addressed in our study. This distinction was necessary to ensure conceptual consistency in applying the TDF to the behaviour of interest. It also reflects the role of MedRec as a foundational safety practice during transitions of care, providing the necessary baseline for other medication-related interventions.

### TDF-informed qualitative study

This report follows the Consolidated criteria for reporting qualitative research (COREQ) (Tong *et al*., 2007) and the Consensus Reporting Items for Studies in Primary Care (CRISP) (Phillips *et al*., 2023) [Supplementary appendix 1]).

#### Participant selection

The preceding quantitative studies (Ascenção *et al.,* 2025), did not suggest any sociodemographic characteristic associated with MedRec practices but anticipated difficulties in the recruitment process for the qualitative study. The low response rate in the quantitative phase likely reflects the high workload in primary care, with multiple competing demands on GPs’ attention and limited time available for research engagement (Morgado *et al*., 2025).

One GP per PHCU was initially contacted based on their interest in the topic or their unit’s experience with MedRec. These purposely selected GPs then invited others, following a snowball-like recruitment strategy.

All participants provided informed consent prior to participation.

#### Setting and data collection

We used focus groups as the primary data collection method.

Focus groups were deliberately composed of GPs from the same PHCU to enhance participation and facilitate in-depth discussions by leveraging their shared professional context. This approach created a safe environment and offered an opportunity to witness the process of collective sense-making on discordant topics.

All focus group discussions were in-person, with audio recordings on two devices to ensure data integrity. The discussions had a maximum duration of 60 minutes.

We used a structured guide (supplementary appendix 2) to direct the discussion, developed based on the TDF and informed by two preceding cross-sectional studies (Ascenção *et al.,* 2025), and selected studies on medication safety (Cadogan *et al*., 2015; Mekonnen *et al*., 2018).

Focus group discussions were conducted after a pilot test until no new relevant information emerged from subsequent sessions.

#### Data analysis

The focus group transcriptions were reviewed manually after automated transcription. All transcripts were imported into MAXQDA 24 (VERBI Software, 2024) for analysis. In the first step, RA mapped segments to the TDF using a deductive approach based on a coding guideline defined and discussed with PB. Coding uncertainties, mostly related to which TDF domain best reflected the participant’s intended meaning, were resolved through discussion between RA and PB. The coding guideline was updated accordingly to ensure consistency across the analysis. A copy of the final coding guideline is available as Supplementary Material. After coding, we revisited the data iteratively to identify themes later classified as perceived barriers or facilitators to MedRec. This approach aligns with the methodology proposed by Atkins *et al.* (2017).

The focus group discussions were conducted in Portuguese. Barriers and facilitators were identified and are reported in English, supported by verbatim translations of illustrative quotes.

#### Research team and reflexivity

A moderator (RA, with short training in qualitative methods) and a co-moderator (PB) conducted the focus group discussions. The researchers’ prior assumptions and professional interests in MedRec were considered in both the design and analysis stages to minimize potential bias. The moderators were mindful of these factors and ensured all participants felt comfortable sharing their views.

### Identification of key behavioural determinants

After the TDF-informed behavioural analysis, we selected the key behavioural determinants (i.e., key TDF domains). The selection of key domains was initially based on the recurrence of barriers/facilitators, the presence of barriers and facilitators in the same domain and strong beliefs affecting behaviour (Atkins *et al*., 2017). Recognizing that barriers/facilitators within one domain often influenced others, we extended our analysis to explore these interconnections.

### Mapping key behavioural determinants to intervention design

The BCW framework recognizes that behaviour results from the dynamic interaction of three core components (capability, opportunity, and motivation), collectively referred to as the COM-B system (Michie *et al*., 2011, 2014). These components can be further mapped onto domains of the TDF, which offers a more granular view (Michie *et al*., 2014). To change behaviour, one or more of these components must be influenced through targeted intervention functions supported by relevant policy categories.

In our work, after key behavioural determinants identification through the TDF, we mapped results to the COM-B system, enabling further mapping to intervention functions through the BCW (Michie *et al*., 2014). Subsequently, we identified the behavioural change techniques (BCT), i.e., the ‘active components of an intervention designed to change behaviour’ (Michie *et al*., 2014) for each intervention function according to the Behaviour Change Technique Taxonomy v1 (Michie *et al*., 2008, 2014). To select the most appropriate BCTs, we started from the list of most frequently used BCTs for each intervention function (Michie *et al*., 2014). Priority was given to BCTs linked to more than one intervention function, which suggested greater theoretical potential to influence behaviour through multiple mechanisms simultaneously.

## Results

We conducted three focus group discussions between February and July 2024. The discussions involved between 6 and 9 GPs per session, for a total of 22 GPs. Demographic characteristics of GPs are presented in Table 1.

**Table 1.** Characteristics of participating GPs in the focus group discussions

	*N*
Gender	
Male	5
Female	17
Age group	
≤ 30 y	7
31–40 y	9
41–50 y	3
51–60 y	3

### Summary of findings from the TDF-informed analysis

#### Knowledge

In the focus group discussions, there was ambiguity in the use of MedRec terminology. At times, discussions uncovered overlapping the MedRec term and other medication optimization practices, including medication review, deprescribing and collecting the best possible medication history (BPMH).

In addition, senior GPs recognized it as a novel term for old practices. On the other hand, junior doctors showed increased familiarity with the concept.

*‘But there wasn’t even the term ‘MedRec’. This is one thing for me that is new.’ (Woman 6, focus group [FG]2)*

*‘In my internship program, this is already included as training (…) there is currently no GP who has at least not studied this subject.’ (Man 1, FG1)*

Transitions of care, namely the first appointment after a hospital discharge, were commonly mentioned. Nevertheless, some GPs advocated that MedRec should be included in every appointment, especially when the setting is favourable (such as during home visits), because medication continuity may be broken without transitions of care (e.g., low adherence).

#### Skills

GPs felt that they learned by example through their supervisors, even though the ‘MedRec’ term was not always explicitly used. The pattern persists when they teach younger GPs.

Clinical practice was regarded as a driver for skills development in MedRec, as prescribing made the need for MedRec obvious.

*‘It’s a process. I don’t think there is ‘I learned this today’ day. I think it is a process being built, and we are building it all our lives.’ (Woman 4, FG3)*

However, GPs feel that skills development, as for knowledge acquisition, should start before the GP medical internship.

*‘(…) if taught in college, perhaps all physicians would be aware of it, whatever their specialty, even at the hospital level.’ (Woman 6, FG2)*

#### Social/Professional role and identity

GPs understand that they are in the right place for MedRec because of the continuity of care they provide and their professional role.

*‘I think there is a perception (…) that we are responsible for patient safety. I think there is a bit of this inherent to our training as family doctors (…).’ (Man 2, FG1)*

However, they recognize MedRec as a responsibility shared by other medical professionals.

#### Beliefs about capabilities, optimism and emotions

GPs reported gaining confidence in MedRec through clinical practice, particularly when observing positive outcomes.

GPs also recognized that changes have been relatively slow in Portuguese primary care. While frustrations were acknowledged, they were attributed more to limitations in the electronic prescribing system than to MedRec itself.

‘What bothers me is that the electronic prescribing system doesn’t work.’ (Woman 2, FG3)

Additionally, structured MedRec practices provided clarity and stability, particularly for GPs with strong attention to detail and a heightened concern for medication accuracy.

#### Social influences

Some suggest that seeing the supervisor undertake MedRec practices is a way to realize that it is an important task to incorporate into daily practice.

#### Behavioural regulation

GPs apply strategies such as dividing consultations, calling patients for an in-person appointment, using quick and targeted questions during consultations, or checking the electronic prescribing system before the appointment to screen for medication changes, mainly when time constraints make a proper MedRec unfeasible.

*‘Sometimes, I can’t address everything within the 20 or 30 minutes available, so I suggest a follow-up consultation to address other issues the patient wants to resolve or even the medication issue if it can wait until another time.’ (Man 1, FG1)*

#### Intentions and goals

GPs clearly intend to enhance MedRec practices, even delaying therapeutic decisions and scheduling additional consultations to collect the BPMH before proceeding.

*‘I often have a lot of difficulty, and I sometimes end up doubling consultations when I realise I want to prescribe something but don’t know what has already been prescribed.’ (Woman 3, FG2)*

Medication management is a broader goal, and deprescribing is mentioned as a strategy to facilitate future MedRec.

#### Environmental context and resources

The electronic prescribing system’s absence of several products, such as over-the-counter medications, negatively impacts the BPMH collection. GPs consistently emphasized that a single, well-integrated platform for electronic prescription, including transitions of care information, could facilitate MedRec.

*‘We can view the last six months, the last 12 months, or the last 18 months in segments, but we don’t have an easy way to see when a medication was introduced, when it was stopped, and whether another medication was started at that time. It becomes very difficult to manage.’ (Woman 3, FG2)*

Nurses working in PCHU and community pharmacies were perceived as key partners for MedRec. On the contrary, GPs do not see pharmacists’ involvement in PHCU teams as universally beneficial.

GPs identified the involvement of multiple prescribers for the same patient and inadequate communication at hospital discharge or after other speciality appointments as key factors increasing the difficulty of performing MedRec.

GPs provided examples of how they involve patients in MedRec practices, specifically when patients are reluctant to engage or unable to recognize medication names or recall their regimens.

*‘And sometimes, this is such a significant obstacle to many appointments that I sometimes repeat and say “look, bring the medicine bag” (…).’ (Woman 6, FG2)*

#### Memory, attention and decision processes

GPs highlighted how the architecture of electronic health records often disrupts their attention and decision-making processes due to fragmented information and make medication changes difficult to register.

*‘Then the aggravating factor is that those who really need MedRec the most are usually the patients whose consultations are already more time-consuming to begin with, even before addressing their medication.’ (Woman 3, FG2)*

Physicians proposed integrating artificial intelligent systems capable of generating real-time alerts during prescribing, while avoiding alert fatigue.

*‘With intelligent software that, as I’m prescribing an ARB [angiotensin receptor blocker], generates an alert that the patient has been prescribed an ACEi [Angiotensin-converting enzyme inhibitor] in the past six months, I think that’s possible through informatics.’ (Woman 1, FG2)*

#### Beliefs about consequences

MedRec was perceived as beneficial for workflow efficiency.

*‘But what I feel is that sometimes losing – not losing, investing! [says another voice], investing this time is time that I won’t waste on other consultations because my job is already easier.’ (Woman 5, FG2)*

GPs recalled specific situations where a lack of coordination in MedRec led to deleterious consequences for patients. There were pressing beliefs about the negative consequences of not engaging in MedRec practices (duplications, omissions, adverse events, drug interactions, and increased costs for the patient), with suggestions that quantifying these outcomes could drive greater investment in MedRec.

#### Reinforcement

Key reinforcing factors included patient recognition, including when MedRec is the primary reason patients actively seek an appointment, the perception of ‘doing the right thing’ and positive outcomes associated with MedRec.

*‘I think just the fact that we can see that (…) the overall quality of the consultation may have improved from one to the next is already a sign, even if the patient doesn’t explicitly say, “Thank you for reconciling my medication.”’ (Man 1, FG1)*

Table 2 summarizes these findings, qualified as barriers and facilitators, with accompanying representative quotes from the focus group discussions.

**Table 2.** Summary of barriers and facilitators for each TDF domain with accompanying representative quotes from the focus group discussions

TDF domain		Influence on behaviour: Barrier (▾)/Facilitator (▴)	Representative quote (participant)
Knowledge		▴ MedRec is already included in the GP training	*In my internship program, (…) there is currently no family doctor who has at least not studied this subject. (Man 1, FG1)*
		▾ Mixed opinions regarding the timing of MedRec	*When we start a follow-up consultation, knowing that other actors have intervened. (Woman 3, GF3)*
		▾ MedRec is a new term for old practices, but explicit mapping has never occurred	*I mean, we would (…) check the medication and see if everything was okay. But there wasn’t even the term’ medication reconciliation.’ This is one thing for me that is new. (Woman 6, FG2)*
		▾ Overlapping with other concepts in medication optimization	*But I think there are levels of reconciliation, that is, within medication reconciliation, I think there are many things, right? (Woman 3, FG1)*
		▾ The label (MedRec) is not explicitly used systematically	*I’m not always thinking that I’m doing medication reconciliation when I’m doing it, most of the time. (Man 1, FG1)*
Skills		▴ Learning through example with supervisors during GP internship	*We learn to do it by example, it is not learned on a course. (Woman 2, FG2)*
		▴ Clinical practice fosters the development of MedRec skills driven by necessity	*I think it’s imperative for anyone working [in primary care] to do it, and inherently, we arrive at it. How can you work without it…? (Woman 3, FG3)*
		▴ Collection of the BPMH is practiced using different sources of information	*(…) if followed up in a hospital (…) I also check the prescriptions, most recent consultations and see if it matches what is prescribed or with what the patient is reporting. (Woman 5, FG2)*
		▾ The label (MedRec) is not explicitly used systematically	*We assume and it is not explicit. Just like it happened to us. (several voices, FG2)*
Social/Professional Role and Identity		▴ MedRec is part of the GP professional role as patient managers, in line with the continuity of care they provide	*(…) they come here proactively to ask for our almost approval to change their medication ‘they changed all my medication, but I’ll come here first and talk to you to see if I can do it’ (Man 1, FG3)*
		▴ MedRec as a patient safety initiative relates to GPs professional attributes	*I think there is a perception (…) that we are responsible for patient safety. I think there is a bit of this here, inherent to our training as family doctors (…) (Man 2, FG1)*
		▾ Responsibility for MedRec should be shared with others	*It should be a shared task with others (…) I think it should involve all the physicians who, in some way, are following that patient, even if it’s for a very specific situation. (Woman 4, FG1)*
Beliefs about capabilities		▴ GPs feel confidence in MedRec skills through practice, particularly when they observe positive results	*The patient is alive, with better feedback, and experiencing fewer side effects. (Several speakers, FG2)*
Optimism		▾ Changes happen but exhibit a slow adoption	*But it has already been very good. We used to be without the EHR (…) (Woman 1, FG3)*
Emotions		▾ Frustration from time-consuming consultations due to inefficiencies in EHR	*And there are some patients we see on the schedule and think, ‘Well, there goes the rest of my morning or afternoon,’ but it is what it is. (Woman 2, FG1)*
Social influences		▴ Role modelling from the supervisor during GP training	*On a course, everyone says what you should do. Now, what if I’ve never seen others do it? It’s because it’s not important, because there’s no time to waste. (Woman 2, FG2)*
Behavioural regulation		▴ Ability to adapt MedRec to specific circumstances.	*Sometimes I can't address everything within the 20 or 30 minutes available, so I suggest a follow-up consultation to address other issues the patient wants to resolve or even the medication issue, if it can wait until another time. (Man 1, FG1)*
Intention		▴ Commitment to optimise MedRec despite daily practice challenges.	*I often have a lot of difficulty, and I sometimes end up doubling consultations when I realise I want to prescribe something but don't know what has already been prescribed. (Woman 3, FG2)*
Goals		▴ MedRec is part of a broader goal of medication management	*The fewer medications the patient is taking, the easier it is, isn’t it? (Woman 7, FG2)*
Environmental context and resources	Electronic health records	▴ There is a system in place for electronic prescriptions	*(…) it makes things easier because we also know the information from others (Woman 4, FG3)*
		▾ The electronic prescribing system is not organized around a medication list but rather prescriptions to be fulfilled	*A single list that all prescribers could update. (Woman 1, FG3)*
		▾ The process of cancelling prescriptions is deferred because of time constraints	*Because no one – I don’t know if you do this here, I don’t have time to do it – goes back to review and cancel all the still-valid prescriptions. (Woman 3, FG2)*
		▾ Several medications/products are absent by default from the electronic prescribing system	*Because what I see here is that my list in the PEM is short. Everything they buy at the pharmacy is much longer. (Woman 3, FG1)*
	MedRec as a multiprofessional process	▴ Nurses working along GPs in PCHUs may flag transitions of care and/or medication changes	*During in-room treatments, they can act as a channel to flag that a consultation is needed to check how things are going, right? (Woman 2, FG3)*
		▴ Pharmacists working in community pharmacies can recognize medication discrepancies or drug interactions that might otherwise go unnoticed	*I think pharmacies should, or I’m not sure if they do, actually, keep a record and alert people, draw attention to it. Because sometimes doctors might not notice, right? (Woman 4, FG3)*
		▾ Lack of broader institutional or shared protocols for MedRec	*Other days it’s a bit exasperating, because there are many more hospital colleagues than there are of us and getting them all to do this might not be so easy. (Man 1, GF1)*
		▾ Inadequate communication (at transitions of care and with other prescribers)	*But I often notice that patients come from the hospital very confused, because they are only told ‘here is your prescription’; they change their medication, and I think sometimes they are too embarrassed to ask, so they don’t, and they end up confused with new prescriptions (…) (Woman 6, FG2)*
	Time	▾ Time constraints and workload	*Either having the availability or not wanting to bother. Or time, so many consultations, so many consultations that there is no time to do it. (Woman 2, FG3)*
	Patient	▾ Patients often lack the health literacy needed to understand the importance of managing their medication regimen	*I, for example, do this; sometimes my colleagues do it too: ‘bring me the boxes of all the medicines you are taking. (Woman 1, FG3)*
Memory, attention and decision processes		▾ Cognitive overload due to fragmented information in the EHR and competing tasks	*It’s not just one task; it’s several. It’s not just one being done at the same time. (several voices, FG2)*
		▾ Absent information relating to previous therapeutic decisions	*And sometimes even we don’t understand why one was stopped and another was started. (Woman 1, FG1)*
Beliefs about consequences		▴ GPs are aware of the scope of positive and negative consequences of MedRec	*I think everyone would benefit from this. I think patients benefit, the system benefits, professionals benefit – in other words, no one loses with this (…) (Woman 2, FG1)*
		▾ There is no indicator to measure the impact of MedRec failures	*Better coding of visits to the emergency department and reconciliation failures. Because perhaps if it were truly well-coded, as they have in the United States, it would be clear that this is a public health issue. Maybe things would move differently. (Woman 3, FG1)(Woman 3, FG1)*
Reinforcement		▴ Patient recognition	*(…) unfortunately, often it’s the patients themselves who ask us, ‘Doctor, I’m very confused about my medication; I need you to help me.’ (Woman 6, FG2)*
		▴ MedRec provides professional fulfilment	*There’s a sense of pride in knowing we’re doing the best for our patient. (Woman 4, FG1)*

BPMH, best possible medication history; EHR, electronic health records – including the electronic prescription system; PCHU, Primary Care Health Unit.

### Key theoretical domains influencing GP-led MedRec

The domain that gathered, by far, the most coded utterances was ‘environmental context and resources’. Coded segments mainly provided beliefs about barriers to MedRec.

In a broader view, however, facilitators in several domains, such as ‘social/professional role’ and ‘beliefs about consequences’, acted as counterbalancing forces that ultimately contributed to the ‘behavioural regulation’ of GPs.

Most of these working forces operated implicitly, as ambiguity regarding what constitutes MedRec often undermines its explicit recognition and GPs view MedRec as part of the GP professional role. Although primarily related to the ‘knowledge’ and ‘skills’ domains, we anticipate that ambiguity has implications across other domains.

As such, three domains emerge as key: ‘knowledge’, ‘skills’ and ‘environmental context and resources’, despite the influence of others.

**Figure 1.**
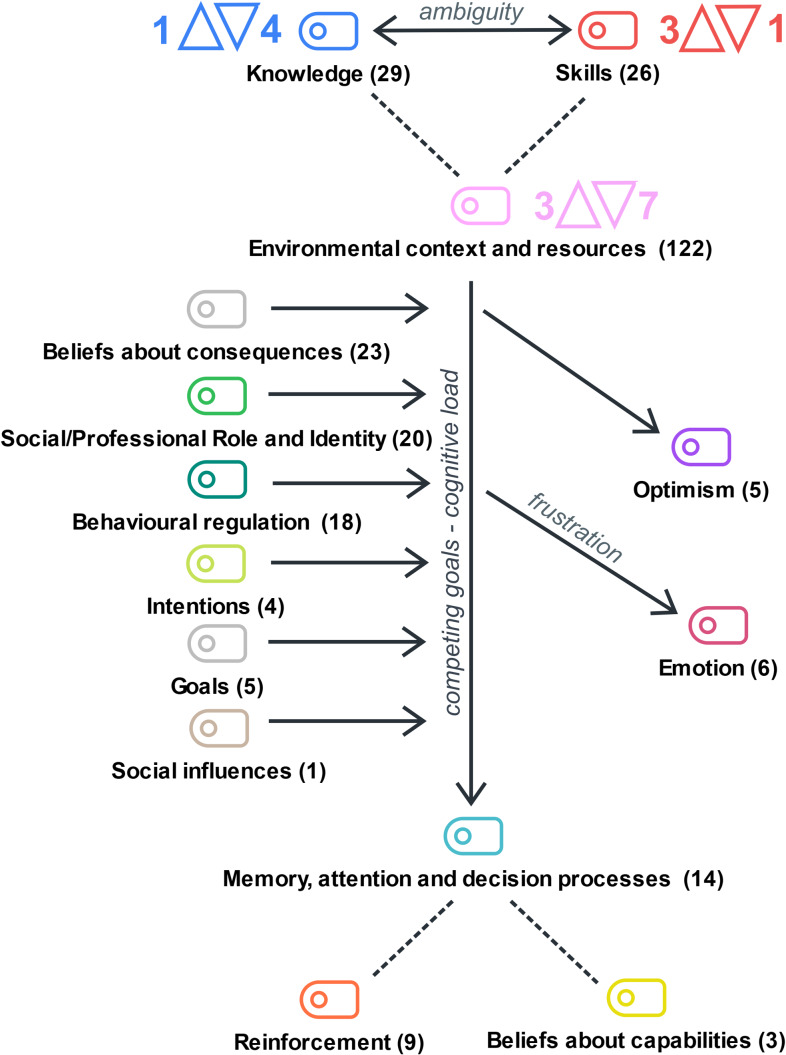
Schematic view of the authors’ interpretation of the interplay of TDF domains for MedRec. TDF theoretical domain codes are displayed along with the number of coded utterances (in brackets). Three domains (‘knowledge’, ‘skills’, and ‘environmental context and resources’) emerged as the most relevant and are characterized by the number of barriers (▾) and facilitators (▴) for each, highlighting their influence on medication reconciliation (MedRec). Barriers in ‘environmental context and resources’ (such as time constraints, inefficient health records, low patient health literacy, and ineffective multi-professional collaboration) can be offset by facilitators in ‘social/professional role’, ‘beliefs about consequences’, and ‘behavioural regulation’. However, barriers persist in ‘memory, attention, and decision processes’, leading to the deprioritization of medRec. These relationships are represented by solid arrows, indicating the cascading effects of barriers and facilitators across domains. Dashed lines represent further connections between domains.

Figure 1 presents a schematic view of the authors’ interpretation of the interplay of these domains.

### Mapping theoretical domains to intervention design using the BCW

The key TDF domains were mapped to the COM-B system and linked to five separate intervention functions, i.e., general types of interventions (Table 3).

**Table 3.** Mapping the key theoretical domains framework identified to the COM-B system and intervention functions

TDF	COM-B	Intervention function
Knowledge	Psychological capability	Education
Skills	Psychological capability	Training
Environmental context and resources	Physical opportunity	Training Restriction Environmental restructuring Enablement

*Note:* informed by the Behaviour Change Wheel framework (Michie *et al*., 2014).

Table 4 presents the most frequently used individual BCTs under these intervention functions. Six BCTs were found to align with more than one intervention function (highlighted in bold) and are proposed to be included in the intervention design due to their greater theoretical breadth and adaptability.

**Table 4.** Mapping between intervention functions and individual behavioural change techniques (BCTs)

Intervention function	Most frequently used individual behavioural change techniques (BCTs)*
Education	Information about social and environmental consequences Information about health consequences **Feedback on behaviour** **Feedback on outcome(s) of the behaviour** **Prompts/cues** **Self-monitoring of behaviour**
Training	Demonstration of the behaviour Instruction on how to perform a behaviour **Feedback on the behaviour** **Feedback on outcome(s) of behaviour** **Self-monitoring of behaviour** Behavioural practice/rehearsal
Restriction	*No BCT in Behaviour Change Technique Taxonomy v1 are linked to this intervention function because they are focused on changing the way that people think, feel and react rather than the way the external environment limits their behaviour*
Environmental restructuring	**Adding objects to the environment** **Prompts/cues** **Restructuring the physical environment**
Enablement	Social support (unspecified) Social support (practical) Goal setting (behaviour) Goal setting (outcome) **Adding objects to the environment** Problem solving Action planning **Self-monitoring of behaviour** **Restructuring the physical environment** Review behaviour goal(s) Review outcome goal(s)

* Six BCTs were found to align with more than one intervention function and are highlighted in bold.

*Note:* informed by the Behaviour Change Technique Taxonomy v1 (Michie *et al.*, 2014).

## Discussion

This qualitative study used the TDF and the BCW to explore behavioural determinants influencing GPs’ MedRec practices. Knowledge and skills inconsistencies, including the implicit framing of MedRec, and environmental barriers, emerged as key challenges, while professional identity acted as a facilitator.

To better understand these findings, it is important to consider that we found that barriers and facilitators often exacerbated each other, a pattern also acknowledged by others (Mather *et al*., 2022) but so far insufficiently described in MedRec.

In particular, our findings suggest that GPs view MedRec as aligned with the core values of Family Practice and the European Definition of General Practice/Family Medicine (Windak *et al*., 2024). These remarks work as a strong facilitator in the ‘Social/Professional Role and Identity’ domain and partly explain the implicit framing of MedRec (i.e., not identifying and naming it as a distinct clinical task), as senior GPs understand that they already practised MedRec when the term was coined. Junior GPs, on the other hand, reveal increased familiarity with the ‘MedRec’ terminology. As such, clinical practice and experience are facilitators in multiple domains (‘Beliefs about Capabilities’, ‘Reinforcement’ and ‘Skills’). In addition, MedRec is subject to contextual learning, and role modelling is an important facilitator in the ‘Social influences’ domain.

Besides this implicit/explicit dichotomy in practice (and training), ambiguity also stemmed from overlapping with other concepts in medication optimization. This is not surprising as MedRec acts as a stepping stone in medication management practices, and there is general agreement that multiple steps can co-occur (reconciliation and review, for instance), particularly when the professional is knowledgeable of the clinical scenario and, concomitantly, the prescriber.

By systematically applying the TDF and mapping key determinants to intervention functions and candidate BCTs through the BCW, our study provides a more structured pathway from behavioural diagnosis to intervention design. ‘Education’ and ‘training’ were suggested via the BCW framework to overcome barriers in the ‘Knowledge’ and ‘Skills’ domains. Possible specific behavioural change techniques would be through ‘Feedback on behaviour’, ‘Feedback on outcome(s) of the behaviour’ and ‘Self-monitoring of behaviour’, which could be introduced both as a process indicator for GPs, but also (at least partially, as for ‘feedback on behaviour’ concerns) during undergraduate training for all medical students. The latter would contribute to the explicit use of MedRec terminology and to present it as an individual clinical task. In addition, at the PHCU level, a team-based, formative audit and feedback approach could be implemented. Each GP could participate in an annual audit of one consultation conducted after a patient’s hospital discharge. These audits would be peer-led and discussed collectively within the PHCU, focusing on whether and how MedRec was carried out, and exploring the clinical relevance and consequences of the actions taken or omitted.

In addition, ‘Prompts/cues’ could be added to the electronic health records, and this is aligned with GPs’ suggestion to create a ‘medication timeline’ with alerts for transitions of care (e.g., hospital discharge). This would also favour the ‘Environmental restructuring’ and ‘enablement’ suggested by the BCW framework as intervention functions. A single platform would also help address the additional complexity derived from multiple prescribers (who, in countries other than Portugal, may include allied health professionals) and reduce the information gap resulting from the lack of data on over-the-counter medications. Such a medication timeline has been suggested by others (Belden *et al*., 2019) to diminish the temporal and cognitive load on physicians. Notwithstanding, this or any other Electronic Medication Reconciliation Tool form should comply with demands such as interoperability, supporting the multiple cognitive strategies applied during MedRec (Bitan *et al*., 2019). Improvement of prescribing systems would benefit communication, as suggested in a qualitative study involving primary health professionals in neighbouring Spain (Rojas-Ocaña *et al*., 2023).

Importantly, both system-wide (such as those related to changes in the electronic health record) and local applications of BCTs should acknowledge and build upon the implicit/explicit dichotomy of MedRec between junior and senior GPs. Specifically, while senior GPs may describe performing MedRec without explicitly labelling it as such, this alone does not constitute evidence of superior knowledge or behaviour. Similarly, familiarity with MedRec terminology among junior GPs does not guarantee consistent practice. Promoting a shared understanding going forward is therefore necessary to overcome implementation barriers. This approach supports behavioural regulation and learning through shared clinical reasoning and aligns with continuous team-based quality improvement practices in primary care.

Our results suggest flexibility in how MedRec is delivered by GPs, attuned to factors such as the type of appointment, reason for seeking care, workload and time constraints. This underscores procedural knowledge and the GPs’ awareness of the specific settings or tools that can facilitate MedRec, identifying facilitators in the ‘Knowledge’ and ‘Behavioural Regulation’ domains. Bitan *et al*. (2019) have previously shown that clinicians apply several cognitive strategies during MedRec, mostly using medical conditions as anchors to which medications are matched, and that over time, experienced clinicians develop intuitions on how to handle challenges, such as the inclusion of new information during the process.

GPs suggested aligning procedures between primary and secondary care. This would facilitate MedRec within PCHUs by reducing ambiguity and streamlining processes. This approach is recommended by the WHO (World Health Organization, 2007) and supported by other studies, where inconsistencies and lack of standardization were also identified as barriers to MedRec (Gionfriddo *et al*., 2021). It could be operationalized by clarifying workflows and information systems (‘Restructuring the physical environment’ BCT), and by introducing standardized tools to support MedRec across care levels (‘Adding objects to the environment’ BCT).

## Strengths and limitations

Our work identified barriers and facilitators that extend and align with previous research (Redmond *et al*., 2020; Gionfriddo *et al*., 2021; Yuan *et al*., 2022), even if not informed by the TDF. In addition, unlike previous studies that typically either do not apply a TDF theoretical lens to study MedRec behaviour (Redmond *et al*., 2020; Gionfriddo *et al*., 2021; Rojas-Ocaña *et al*., 2023) or identify a large number of relevant but separate TDF domains influencing GP practices (Cadogan et al., 2015), we adopted an approach aimed at a deeper understanding of GPs’ practices and how TDF domains interact to produce behaviour. In doing so, we were able to contribute to the theory-informed development of a MedRec intervention at a local level and confidently propose implementation strategies to be tested in future studies. Conducting research at a local level remains essential, as regional idiosyncrasies may influence the implementation and effectiveness of MedRec, highlighting the need for context-specific studies. Our work demonstrates that this is an attainable objective. On the other hand, by employing internationally recognized theoretical frameworks, the findings presented here may be transferable and informative to other health systems seeking to strengthen GP-led MedRec practices through behavioural change.

Our methodological decisions, taken to ensure that the research was feasible, may have introduced biases. First, although data from our focus group discussions provided rich insights, recruiting participants through purposive sampling followed by snowballing may have introduced selection bias, favouring GPs who are more interested in MedRec or more engaged in reflective practice. Furthermore, conducting focus group discussions within the same PHCU may have introduced conformity bias, and findings may not fully capture the diversity of practices across the LVT Region or the Portuguese NHS. However, bringing together GPs from the same PHCU facilitated focus group scheduling and created a safe environment between junior and senior clinicians, enabling discussions grounded in shared day-to-day experience. By informing the data collection with insights from a preceding quantitative phase, including two cross-sectional studies, we believe this approach enhanced the trustworthiness of our analysis and contributed to achieving data saturation after three focus group discussions. For example, since few units reported having formal MedRec procedures in place, it is less likely that local formal procedures alone shaped participants’ perspectives. Secondly, our qualitative analysis involved a single primary coder, supported by regular discussions with a second researcher. Using a clearly defined coding guideline and a coherence check of the findings by a third researcher helped mitigate potential individual subjectivity.

While our work sheds light on the role of GPs in MedRec, we acknowledge that, from a system point of view, MedRec in Primary Care depends critically on what happens in other levels and contexts of care, including a possible spillover effect from and onto other patient safety interventions. Strategies to optimize collaborative work while avoiding responsibility diffusion should also be encouraged. Patient empowerment, either through health literacy initiatives or during contact visits, should also be sought.

## Implications for research, clinical practice and medical education

While this study provides a theory-informed foundation for the design of implementation strategies to improve MedRec practices, future research should address the appropriateness of the proposed behaviour change techniques and the most feasible and acceptable modes of delivery. Certain components of the proposed intervention could be more pragmatically introduced through organizational adjustments at the PHCU or regional level. In contrast, others, particularly those related to digital infrastructure development, would require central policy support and investment. Given the resource constraints in primary care settings, future work should ensure that intervention development remains pragmatic and that existing infrastructures are leveraged whenever possible rather than assuming substantial additional investments. This is even more important as previous research remains conflicting regarding MedRec’s effectiveness on patient-relevant outcomes (Guisado-Gil *et al*., 2020) and its optimal integration with broader medication optimization strategies (Stuijt *et al*., 2022). Effectiveness-implementation Hybrid Designs could address these research questions.

Lastly, concerning ramifications beyond the direct scope of primary care, we emphasize the need to address the gap in MedRec undergraduate medical training while revising the European core curriculum of medical students (Tichelaar *et al*., 2020).

## Conclusions

This qualitative study explored the barriers and facilitators influencing MedRec implementation in Portugal’s largest primary care region through a TDF-informed lens. By exploring the interconnection between identified barriers and facilitators across multiple TDF domains, we suggest that ‘knowledge’, ‘skills’ and ‘environmental context and resources’ are key determinants of effective MedRec implementation. We expand on previous studies in primary care by proposing candidate BCTs informed by the BCW framework that should be combined with appropriate modes of delivery and further analysed to bridge the gap between policy and MedRec practice pragmatically.

## Supporting information

10.1017/S1463423626101200.sm001 Ascenção et al. supplementary material Ascenção et al. supplementary material

## Data Availability

Data are available from the corresponding author upon reasonable request. Each request will be considered individually and granted only if compliant with ethical guidelines and institutional data protection regulations.

## Supplementary material

The supplementary material for this article can be found at https://doi.org/10.1017/S1463423626101200.
