# Supplementary material for: Supporting medication reconciliation in primary care: a theory-informed qualitative study in Portugal
Source: Prim Health Care Res Dev. 2026 May 6;27:e54. doi: 10.1017/S1463423626101200 (PMC13161821; doi:10.1017/S1463423626101200)
Supplement: Ascenção et al. supplementary material [file S1463423626101200sup001.docx]

**SUPPLEMENTARY APPENDIX 1**

“**Supporting medication reconciliation in general practice: a theory-informed qualitative study in Portugal**”

**Consolidated criteria for reporting qualitative studies (COREQ): 32-item checklist** (Adapted from Tong et al. 2007)

| **Item** | **Report on page no.; section/subsection** |
| --- | --- |
| **Domain 1: Research team and reﬂexivity** | |
| *Personal Characteristics* | |
| 1. Interviewer/facilitator | 8; “Research Team and Reflexivity” |
| 2. Credentials | 8; “Research Team and Reflexivity” |
| 3. Occupation | 8; “Research Team and Reflexivity” |
| 4. Gender | 8; “Research Team and Reflexivity” |
| 5. Experience and training | 8; “Research Team and Reflexivity” |
| *Relationship with participants* | |
| 6. Relationship established | 8; “Research Team and Reflexivity” |
| 7. Participant knowledge of the interviewer | 8; “Research Team and Reflexivity” |
| 8. Interviewer characteristics | 8; “Research Team and Reflexivity” |
| **Domain 2: study design** | |
| *Theoretical framework* | |
| 9. Methodological orientation and Theory | 4; “Methods” |
| *Participant selection* | |
| 10. Sampling | 5-6; “Participant Selection” |
| 11. Method of approach | 5-6; “Participant Selection” |
| 12. Sample size | 8; “Results” |
| 13. Non-participation | Not applicable |
| *Setting* | |
| 14. Setting of data collection | 5-6; “Setting and Data Collection” |
| 15. Presence of non-participants | Inferred on page 6, non-participants were not present |
| 16. Description of sample | 9; “Results” |
| *Data collection* | |
| 17. Interview guide | Included as a supplementary file; pilot tested |
| 18. Repeat interviews | Not applicable |
| 19. Audio/visual recording | 7; “Setting and Data Collection” |
| 20. Field notes | Yes during the focus group, not reported |
| 21. Duration | 7; “Setting and Data Collection” |
| 22. Data saturation | 7; “Setting and Data Collection” |
| 23. Transcripts returned | No |
| **Domain 3: analysis and ﬁndings** | |
| *Data analysis* | |
| 24. Number of data coders | 7-8; “Data analysis” |
| 25. Description of the coding tree | 7-8; “Data analysis” |
| 26. Derivation of themes | 7-8; “Data analysis” |
| 27. Software | 7-8; “Data analysis” |
| 28. Participant checking | No |
| *Reporting* | |
| 29. Quotations presented | 9-15; “Results” and Table 2 |
| 30. Data and ﬁndings consistent | 15-19; “Discussion” |
| 31. Clarity of major themes | 15-19; “Discussion” |
| 32. Clarity of minor themes | 15-19; “Discussion” |

**Consensus Reporting Items for Studies in Primary Care (CRISP)** (Adapted from Phillips et al. 2023)

| **Reporting Item - 1** | **Included?** | | | **Section*** | **Notes** |
| --- | --- | --- | --- | --- | --- |
|  | **Y** | **N** | **N/A** |  |  |
| **1. Include “primary care” and/or discipline-specific terms in the title, abstract, and/or key words.** | Y |  |  | I | Included in the title, abstract, and key words. |

| **Reporting Item - 2** | **Included?** | | | **Section*** | **Notes** |
| --- | --- | --- | --- | --- | --- |
|  | **Y** | **N** | **N/A** |  |  |
| **2. Describe the study rationale and importance for primary care.** | | | | | |
| **2a.** Explain the rationale for the research question and how it relates to primary care. | Y |  |  | I |  |
| **2b.** Describe the importance or relevance of the topic under study in the primary care setting. | Y |  |  | I |  |
| **2c.** Identify any theory, model, or framework used, and explain why it is appropriate to the research question in primary care. | Y |  |  | I |  |

| **Reporting Item - 3** | **Included?** | | | **Section*** | **Notes** |
| --- | --- | --- | --- | --- | --- |
|  | **Y** | **N** | **N/A** |  |  |
| **3. Describe the research team’s primary care experience and collaboration.** | | | | | |
| **3a.** Describe the research team’s expertise and experience in primary care practice and/or research. | Y |  |  | M |  |
| **3b.** Describe whether and how primary care patients, practicing clinicians, community members, or other stakeholders were involved in the research process. | Y |  |  | M |  |

| **Reporting Item - 4** | **Included?** | | | **Section*** | **Notes** |
| --- | --- | --- | --- | --- | --- |
|  | **Y** | **N** | **N/A** |  |  |
| **4. Describe the study participants and populations in the context of primary care.** | | | | | |
| **4a.** Use person-focused language to refer to the research populations and participants, or use terms based on patient preferences | Y |  |  | R |  |
| **4b.** If reporting personal characteristics of participants, report the source of the data, the rationale for using it, and the rationale for any classifications used. | Y |  |  | R |  |
| **4c.** Describe the participants and populations in sufficient detail to allow comparison to other primary care patient populations. |  |  | N/A | R |  |
| **4d.** Specify whether participants have pre-existing therapeutic relationships with the clinical team or are new patients. |  |  | N/A | M/R |  |

| **Reporting Item - 5** | **Included?** | | | **Section*** | **Notes** |
| --- | --- | --- | --- | --- | --- |
|  | **Y** | **N** | **N/A** |  |  |
| **5. Describe the conditions under study in the context of primary care.** | | | | | |
| **5a.** Describe whether the condition under study is acute or chronic. |  |  | N/A | M/R |  |
| **5b.** Report how multimorbidity is considered and how it might affect interpretation of the study findings/ results. |  |  | N/A | M |  |

| **Reporting Item - 6** | **Included?** | | | **Section*** | **Notes** |
| --- | --- | --- | --- | --- | --- |
|  | **Y** | **N** | **N/A** |  |  |
| **6. Describe the clinical encounter under study in the context of primary care.** | | | | | |
| **6a.** Specify whether the study focus is an isolated clinical encounter or a longitudinal course of care. If it is an isolated clinical encounter, specify whether it is the first visit or a follow-up visit for the condition under study |  |  | N/A | M |  |

| **Reporting Item - 7** | **Included?** | | | **Section*** | **Notes** |
| --- | --- | --- | --- | --- | --- |
|  | **Y** | **N** | **N/A** |  |  |
| **7. Describe the patient care team.** | | | | | |
| **7a.** If care is delivered by teams, describe the team members and their roles. |  |  | N/A | R |  |
| **7b.** For each clinician category, report profession, specialty, and qualifications. |  |  | N/A | R |  |

| **Reporting Item - 8** | **Included?** | | | **Section*** | **Notes** |
| --- | --- | --- | --- | --- | --- |
|  | **Y** | **N** | **N/A** |  |  |
| **8. Describe the study interventions in the context of primary care.** | | | | | |
| **8a.** Describe interventions and their implementation in sufficient detail to enable the reader to assess applicability in their own setting. | **Y** |  |  | M |  |
| **8b.** Describe any clustering or grouping of patients, participants, clinicians, teams, or practices, and how it was addressed in the analysis. |  |  | N/A | M/R |  |
| **8c.** Describe the health care system in sufficient detail to allow comparisons to other systems. | **Y** |  |  | I/D | Included in the Methods, under “Study setting”. |

| **Reporting Item - 9** | **Included?** | | | **Section*** | **Notes** |
| --- | --- | --- | --- | --- | --- |
|  | **Y** | **N** | **N/A** |  |  |
| **9. Describe study measures used and their relevance to primary care.** | | | | | |
| **9a.** Report whether study measurement tools have been validated in primary care populations or settings. |  |  | N/A | M |  |
| **9b.** Describe how the measurement tools used are meaningful to primary care patients and their care. |  |  | N/A | M |  |
| **9c.** Report findings/results to be clinically interpretable by primary care clinicians and patients. |  |  | N/A | R |  |

| **Reporting Item - 10** | **Included?** | | | **Section*** | **Notes** |
| --- | --- | --- | --- | --- | --- |
|  | **Y** | **N** | **N/A** |  |  |
| **10. Discuss the meaning of study findings/results in the context of primary care.** | | | | | |
| **10a.** Discuss implications of the study findings/results for research, patient care, education, and policy with specific focus on primary care. | Y |  |  | D |  |
| **10b.** Discuss the implications of study recommendations on demands and priorities in primary care practice. | Y |  |  | D |  |
| **10c.** Comment on any research processes that might influence the applicability of the study findings/results in diverse primary care settings. | Y |  |  | D |  |

***Section:** I = Introduction, M = Method, R = Results, D = Discussion

**SUPPLEMENTARY APPENDIX 2**

**Focus Group Guide**

| **TDF Domain** | **Questions (in Portuguese, as used in our study)** | **Questions (in English)** |
| --- | --- | --- |
| **Knowledge**  (An awareness of the existence of something) | **É possível realizar a RecMed sem ter conhecimento prévio sobre este termo?**  .. sem saber a definição? Fazem mas não lhe põem o nome?  *Como saber se sabemos RecMed aos mesmos processos?*  Haverá um momento-chave para realizar RecMed em MGF? | **Is it possible to carry out MedRec without prior knowledge of the term?**  … without knowing the definition? Do you do it but not name it as such?  *How can we know if we refer to the same processes when we talk about MedRec?*  Is there a key moment to carry out MedRec in Family Medicine? |
| **Goals**  (Mental representations of outcomes or end states that an individual wants to achieve) | **A RecMed é um objetivo da prática da MGF?** | Is MedRec a goal of Family Medicine practice? |
| **Social/Professional Role and Identity**  (A coherent set of behaviours and displayed personal qualities of na individual in a social or work setting) | **A realização da RecMed faz parte do papel dos MF?**  É possível ser MF sem fazer RecMed? Ou pode ser feito por outro? | **Is performing MedRec part of the role of GPs?**  Is it possible to be a GP without doing MedRec? Or can it be done by someone else? |
| **Skills**  (An ability or proficiency acquired through practice) | **Que parte da formação do MF é mais útil para o desenvolvimento destas competências?** | Which part of GP training is most useful for developing these skills? |
| **Beliefs about Capabilities**  (Acceptance of the truth, reality, or validity about an ability, talent, or facility that a person can put to constructive use) | **Quão confiantes se sentem na realização desta tarefa?** | How confident do you feel in performing this task? |
| **Emotion**  (A complex reaction pattern, involving experiential, behavioural, and physiological elements, by which the individual attempts to deal with a personally significant matter or event) | **O que sentem?**  … a pessoa pode sentir-se capaz e mesmo assim «não gostar» | **What do you feel?**  … a person may feel capable and still “not like” doing it |
| **Social influences**  (Those interpersonal processes that can cause individuals to change their thoughts, feelings, or behaviours) | **É valorizado o papel dos MF na RecMed?** | Is the role of GPs in MedRec valued? |
| **Memory, Attention and Decision Processes**  (The ability to retain information, focus selectively on aspects of the environment and choose between two or more alternatives) | **Quão fácil é recordar o papel da RecMed durante uma consulta ou antes de uma prescrição?** | How easy is it to recall the role of MedRec during a consultation or before prescribing? |
| **Environmental Context and Resources**  (Any circumstance of a person's situation or environment that discourages or encourages the development of skills and abilities, independence, social competence, and adaptive behaviour) | **Há os recursos necessários?**  O contexto ajuda ou prejudica a prática da RecMed?  … questões que ajudem/prejudiquem, por exemplo, a lista de utentes | **Are the necessary resources available?**  Does the context support or hinder the practice of MedRec?  … factors that may help/hinder, for example, the patient list |
| **Reinforcement**  (Increasing the probability of a response by arranging a dependent relationship, or contingency, between the response and a given stimulus) | **Existe algum reforço positivo/estímulo à prática da RecMed em CSP?** | **Is there any positive reinforcement or stimulus for the practice of MedRec in Primary Health Care?** |
| **Beliefs about Consequences**  (Acceptance of the truth, reality, or validity about outcomes of a behaviour in a given situation) | **Quais são as consequências de não realizar RecMed?**  Para o médico? Para o doente? Para o sistema de saúde?  Os benefícios compensam o tempo? | **What are the consequences of not doing MedRec?**  For the physician? For the patient? For the health system?  Do the benefits outweigh the time required? |
| **Intentions**  (A conscious decision to perform a behaviour or a resolve to act in a certain way) | **Estão motivados para realizar RecMed?** | **Are you motivated to carry out MedRec?** |
| **Optimism**  (The confidence that things will happen for the best or that desired goals will be attained) | **A RecMed algum dia será prática em todas consultas e para todos os doentes?** | **Will MedRec ever become routine in all consultations and for all patients?** |
| **Behavioural Regulation**  (Anything aimed at managing or changing objectively observed or measured actions) | **Receberam feedback de outros profissionais relativamente à RecMed?**  Ensinam aos internos de formação específica?  … se não tiverem conhecimento prévio RecMed – como se pode ensinar? | **Have you received feedback from other professionals regarding MedRec?**  Do you teach it to Family Medicine trainees?  … if there is no prior knowledge of MedRec – how can it be taught? |

MF, médico de família. RecMed, reconciliação da medicação.

GP, general practitioner. MedRec, medication reconciliation.

**SUPPLEMENTARY APPENDIX 3**

**Coding guideline**

| **Domain** | **Constructs and definitions**  **(Cane et al, 2012; doi:10.1186/1748-5908-7-37)** | **Additional guidance for appropriate coding** |
| --- | --- | --- |
| **1. Knowledge** | **Knowledge (including knowledge of condition /scientific rationale)** - An awareness of the existence of something.  **Procedural knowledge** - Knowing how to do something.  **Knowledge of task environment** - Knowledge of the social and material context in which a task is undertaken. | - Code here when the GP refers to what they know about MedRec, including definitions, purpose, rationale or context in which it should be applied. - Include references to training only if they contribute directly to knowledge acquisition. - Prioritise this domain when the emphasis is on awareness or understanding, rather than on execution or confidence. |
| **2. Skills** | **Skills** - An ability or proficiency acquired through training and/or practice.  **Skills development** - The gradual acquisition or advancement through progressive stages of an ability or proficiency acquired through training and practice.  **Competence** - One’s repertoire of skills, and ability especially as it is applied to a task or set of tasks.  **Ability** - Competence or capacity to perform a physical or mental act. Ability may be either unlearned or acquired by education and practice.  **Interpersonal skills** - An aptitude enabling a person to carry on effective relationships with others, such as an ability to cooperate, to assume appropriate social responsibilities or to exhibit adequate flexibility.  **Practice** - Repetition of an act, behaviour, or series of activities, often to improve performance or acquire a skill.  **Skill assessment** - A judgment of the quality, worth, importance, level, or value of an ability or proficiency acquired through training and practice. | - Focus on the technical skills and specific competencies that are needed to conduct MedRec. - Focus on clinical practice and how MedRec is performed according to practical and technical skills. - Code here when describing the practical aspects of MedRec. - Code training aspects here, if a way to develop abilities. |
| **3. Social/Professional Role and Identity** | **Professional identity** - The characteristics by which an individual is recognised relating to, connected with or befitting a particular profession.  **Professional role** - The behaviour considered appropriate for a particular kind of work or social position.  **Social identity** - The set of behavioural or personal characteristics by which an individual is recognisable [and portrays] as a member of a social group.  **Identity** - An individual’s sense of self defined by a) a set of physical and psychological characteristics that is not wholly shared with any other person and b) a range of social and interpersonal affiliations (e.g., ethnicity) and social roles.  **Professional boundaries** - The bounds or limits relating to, or connected with a particular profession or calling.  **Professional confidence** - An individual’s belief in his or her repertoire of skills, and ability especially as it is applied to a task or set of tasks.  **Group identity** - The set of behavioural or personal characteristics by which an individual is recognisable [and portrays] as a member of a group.  **Leadership** - The processes involved in leading others, including organising, directing, coordinating and motivating their efforts toward achievement of certain group or organisation goals.  **Organisational commitment** - An employee’s dedication to an organisation and wish to remain part of it. Organisational commitment is often described as having both an emotional or moral element and a more prudent element. | - Family Medicine identity. - Code here when GPs describe practical abilities or techniques used to conduct MedRec (e.g., checking lists, interpreting prescriptions, managing discrepancies). - Include references to training only when described as skill-building. |
| **4. Beliefs about Capabilities** | **Self-confidence** - Self-assurance or trust in one’s own abilities, capabilities and judgment.  **Perceived competence -** An individual’s belief in his or her ability to learn and execute skills.  **Self-efficacy** - An individual’s capacity to act effectively to bring about desired results, as perceived by the individual.  **Perceived behavioural control** - An individual’s perception of the ease or difficulty of performing the behaviour of interest.  **Beliefs** - The thing believed; the proposition or set of propositions held true.  **Self-esteem** - The degree to which the qualities and characteristics contained in one’s self- concept are perceived to be positive.  **Empowerment** - The promotion of the skills, knowledge and confidence necessary to take great control of one’s life as in certain educational or social schemes; the delegation of increased decision-making powers to individuals or groups in a society or organisation.  **Professional confidence** - An individual’s belief in his or her repertoire of skills, and ability especially as it is applied to a task or set of tasks. | - Code here when GPs reflect on their confidence to carry out MedRec. - Code here when GPs address their capability to apply what they know/learn (are they capable?). |
| **5. Optimism** | **Optimism** - The attitude that outcomes will be positive and that people’s wishes or aims will ultimately be fulfilled.  **Pessimism** - The attitude that things will go wrong and that people’s wishes or aims are unlikely to be fulfilled.  **Unrealistic optimism** - The inert tendency for humans to over-rate their own abilities and chances of positive outcomes compared to those of other people.  **Identity** - An individual’s sense of self defined by a) a set of physical -and psychological characteristics that is not wholly shared with any other person and b) a range of social and interpersonal affiliations (e.g., ethnicity) and social roles. | - Code here when debating whether the implementation problems will ever be solved. |
| **6. Beliefs about Consequences** | **Beliefs** - The thing believed; the proposition or set of propositions held true.  **Outcome expectancies** - Cognitive, emotional, behavioural, and affective outcomes that are assumed to be associated with future or intended behaviours. These assumed outcomes can either promote or inhibit future behaviours.  **Characteristics of outcome expectancies** - Characteristics of the cognitive, emotional and behavioural outcomes that individuals believe are associated with future or intended behaviours and that are believed to either promote or inhibit these behaviours. These include whether they are sanctions/rewards, proximal/distal, valued/not valued, probable/improbable, salient/not salient, perceived risks or threats.  **Anticipated regret** - A sense of the potential negative consequences of a decision that influences the choice made: for example an individual may decide not to make an investment because of the feelings associated with an imagined loss.  **Consequents** - An outcome of behaviour in a given situation. | - Code here when addressing the consequences of conducting MedRec or not. - Code here if GPs discuss the perception of these consequences. |
| **7. Reinforcement** | **Rewards (proximal / distal, valued / not valued, probable / improbable)** - Return or recompense made to, or received by a person contingent on some performance.  **Incentives** - An external stimulus, such as condition or object, that enhances or serves as a motive for behaviour.  **Punishment** - The process in which the relationship between a response and some stimulus or circumstance results in the response becoming less probable; a painful, unwanted or undesired event or circumstance imposed as a penalty on a wrongdoer.  **Consequents** - An outcome of behaviour in a given situation.  **Reinforcement** - A process in which the frequency of a response is increased by a dependent relationship or contingency with a stimulus.  **Contingencies** - A conditional probabilistic relation between two events. Contingencies may be arranged via dependencies or they may emerge by accident.  **Sanctions** - A punishment or other coercive measure, usually administered by a recognised authority, that is used to penalise and deter inappropriate or unauthorised actions. | - Code here for the perception of incentives. |
| **8. Intentions** | **Stability of intentions** - Ability of one’s resolve to remain in spite of disturbing influences.  **Stages of change model** - A model that proposes that behaviour change is accomplished through five specific stages: Pre-contemplation, Contemplation, Preparation, Action, and Maintenance.  **Transtheoretical model and stages of change** - A five-stage theory to explain changes in people’s health behaviour. It suggests that change takes time, that different interventions are effective at different stages, and that there are multiple outcomes occurring across the stages. | - Code here the plan for conducting MedRec, and whether specific plans are in place. - Code if an internal commitment to practice MedRec is present or debated. - Different from Goals. |
| **9. Goals** | **Goals (distal / proximal)** - Desired state of affairs of a person or system, these may be closer (proximal) or further away (distal).  **Goal priority** - Order of importance or urgency of end states toward which one is striving.  **Goal / target setting** - A process that establishes specific time based behaviour targets that are measurable, achievable and realistic.  **Goals (autonomous / controlled)** - The end state toward which one is striving: the purpose of an activity or endeavour. It can be identified by observing that a person ceases or changes its behaviour upon attaining this state; proficiency in a task to be achieved within a set period of time.  **Action planning** - The action or process of forming a plan regarding a thing to be done or a deed.  **Implementation intention** - The plan that one creates in advance of when, where and how one will enact a behaviour. | - Code here if the discussion is on the extent of the wish to conduct MedRec. - Code here when plans and outcomes are set and defined in specific terms in the present time. |
| **10. Memory, Attention and Decision Processes** | **Memory** - The ability to retain information or a representation of a past experience, based on the mental processes of learning or encoding retention across some interval of time, and retrieval or  reactivation of the memory; specific information of a specific  past.  **Attention** - A state of awareness in which the senses are focussed selectively on aspects of the environment and the central nervous system is in a state of readiness to respond to stimuli.  **Attention control** - The extent to which a person can concentrate on relevant cues and ignore all irrelevant cues in a given situation.  **Decision making** - The cognitive process of choosing between two or more alternatives, ranging from the relatively clear cut to the complex.  **Cognitive overload / tiredness** - The situation in which the demands placed on a person by mental work are greater than a person’s mental abilities. | - Use this domain when the utterance highlights cognitive demands, lack of attention, or difficulty making decisions about MedRec due to information overload or system architecture. - Include references to digital tools if described as supporting cognitive processes (e.g., memory triggers, alerts). - Prioritise this domain over “Environmental context and resources” when the issue is mental workload, not system availability. |
| **11. Environmental Context and Resources** | **Environmental stressors** - External factors in the environment that cause stress.  **Resources / material resources** - Commodities and human resources used in enacting a behaviour.  **Organisational culture /climate** - A distinctive pattern of thought and behaviour shared by members of the same organisation and reflected in their language, values, attitudes, beliefs and customs.  **Salient events / critical incidents** - Occurrences that one judges to be distinctive, prominent or otherwise significant.  **Person x environment interaction** - Interplay between the individual and their surroundings.  **Barriers and facilitators** - In psychological contexts barriers/facilitators are mental, emotional or behavioural limitations/strengths in individuals or groups. | - Code when naming physical and human resources that could support/hinder MedRec. - Also consider the patient, including patient empowerment as a resource that influences MedRec. |
| **12. Social influences** | **Social pressure** - The exertion of influence on a person or group by another person or group.  **Social norms** - Socially determined consensual standards that indicate a) what behaviours are considered typical in a given context and b) what behaviours are considered proper in the context.  **Group conformity** - The act of consciously maintaining a certain degree of similarity to those in your general social circles.  **Social comparisons** - The process by which people evaluate their attitudes, abilities, or performance relative to others.  **Group norms** - Any behaviour, belief, attitude or emotional reaction held to be correct or acceptable by a given group in society.  **Social support** - The apperception or provision of assistance or comfort to others, typically in order to help them cope with a variety of biological, psychological and social stressors. Support may arise from any interpersonal relationship in an individual’s social network, involving friends, neighbours, religious institutions, colleagues, caregivers or support groups.  **Power** - The capacity to influence others, even when they try to resist this influence.  **Intergroup conflict** - Disagreement or confrontation between two or more groups and their members. This may involve physical violence, interpersonal discord, or psychological tension.  **Alienation** - Estrangement from one’s social group; a deep seated sense of dissatisfaction with one’s personal experiences that can be a source of lack of trust in one’s social or physical environment or in oneself; the experience of separation between thoughts and feelings.  **Group identity** - The set of behavioural or personal characteristics by which an individual is recognisable [and portrays] as a member of a group.  **Modelling** - In developmental psychology the process in which one or more individuals or other entities serve as examples (models) that a child will copy. | - Code the supervisor’s influence here only if it is considered pressure from an authoritative figure. - Social influence can arise from other professions as well. |
| **13. Emotion** | **Fear** - An intense emotion aroused by the detection of imminent threat, involving an immediate alarm reaction that mobilises the  organism by triggering a set of physiological changes.  **Anxiety** - A mood state characterised by apprehension and somatic symptoms of tension in which an individual anticipates  impending danger, catastrophe or misfortune.  **Affect** - An experience or feeling of emotion, ranging from suffering to elation, from the simplest to the most complex sensations of feelings, and from the most normal to the most pathological emotional reactions.  **Stress** - A state of physiological or psychological response to internal or external stressors.  **Depression** - A mental state that presents with depressed mood, loss of interest or pleasure, feelings of guilt or low self-worth, disturbed sleep or appetite, low energy, and poor concentration.  **Positive / negative affect** - The internal feeling/state that occurs when a goal has/has not been attained, a source of threat has/has not been avoided, or the individual is/is not satisfied with the present state of affairs.  **Burn-out** - Physical, emotional or mental exhaustion, especially in one’s job or career, accompanied by decreased motivation, lowered performance and negative attitudes towards oneself and others. | - Code here if there is a perception of an emotional response from MedRec. |
| **14. Behavioural Regulation** | **Self-monitoring** - A method used in behavioural management in which individuals keep a record of their behaviour, especially in connection with efforts to change or regulate the self; a personality trait reflecting an ability to modify one’s behaviour in response to situation.  **Breaking habit** - To discontinue a behaviour or sequence of behaviours that is automatically activated by relevant situational cues.  **Action planning** - The action or process of forming a plan regarding a thing to be done or a deed. | - Code here when there are strategies and systems in place to monitor and manage MedRec. - If feedback was received, was it useful to change behaviour? - Code here when addressing self-monitoring (either frequency, response to barriers or the establishment of routines or habits to help MedRec). |
